# Supplementary material for: A New Perspective on SPME and SPME Arrow: Formaldehyde Determination by On-Sample Derivatization Coupled with Multiple and Cooling-Assisted Extractions
Source: Molecules. 2023 Jul 16;28(14):5441. doi: 10.3390/molecules28145441 (PMC10383053; doi:10.3390/molecules28145441)

SUPPLEMENTARY MATERIALS

A NEW PERSPECTIVE ON SPME AND SPME ARROW: FORMALDEHYDE DETERMINATION BY ON-SAMPLE DERIVATIZATION COUPLED WITH MULTIPLE AND COOLING ASSISTED EXTRACTIONS.

Stefano Dugheri[a]*, Giovanni Cappelli[b], Niccolò Fanfani[c], Jacopo Ceccarelli[b], Giorgio Marrubini[d], Donato Squillaci[b], Veronica Traversini[b], Riccardo Gori[e], Nicola Mucci[b] and Giulio Arcangeli[b]

[a]Industrial Hygiene and Toxicology Laboratory, University Hospital Careggi, Florence 50139, Italy

[b]Department of Experimental and Clinical Medicine, University of Florence, Florence 50134, Italy

[c]Department of Experimental and Clinical Biomedical Sciences "Mario Serio", University of Florence, Florence 50134, Italy

[d]Department of Drug Sciences, University of Pavia, Pavia, 27100, Italy

[e]Department of Civil and Environmental Engineering, University of Florence, Florence, 50139, Italy

*stefano.dugheri@unifi.it

Design of Experiments optimization

The cooling-assisted extraction step was also studied using a Design of Experiments approach to assess the best conditions for derivatizing and sampling FA using a conventional SPME and the SPME Arrow.

The response selected was the area of the FA peak in the chromatograms, and the study's goal was to maximize the response. The design selected was a full factorial 2^3, including eight extractions with each fiber plus three additional independent extractions to test the models' reliability in prediction. The test extractions were carried out in the center of the experimental domain, defined by the three ranges of the factors under examination.

Table S1 reports the factors studied, their ranges, the experimental plan, the experimental matrix, and the results obtained, while Equations S1 and S2 report the model equations obtained for SPME and SPME Arrow, respectively.

Table S1 – Design of the cooling-assisted extraction experiment with SPME and SPME Arrow

Factors and abbreviations	Levels		
	Minimum	Central	Maximum
Exposure Temperature, T_{exp} (°C)	10	15	20
Sampling Temperature, T_{sampl} (°C)	60	70	80
Exposure time, time (min)	15	22.5	30

T_{exp} (°C)	T_{sampl} (°C)	time (min)	T_{exp}	T_{sampl}	time	SPME	SPME Arrow
10	80	30	-1	1	1	5041699	14559691
20	80	30	1	1	1	4552738	11679758
10	60	30	-1	-1	1	4741106	13130552
20	60	30	1	-1	1	4408915	9388926
10	80	15	-1	1	-1	4853022	13606461
20	80	15	1	1	-1	4516790	11184696
10	60	15	-1	-1	-1	4688216	12026822
20	60	15	1	-1	-1	4319859	9370726

$$A_{SPME} = 4640293 - 190718 \times T_{exp} - 100769 \times T_{sampl} - 45821 \times time + 15580 \times T_{exp} \times T_{sampl} + 14570 \times T_{exp} \times time + 10335 \times T_{exp} \times time - 23612 \times T_{exp} \times T_{sampl} \times time$$

Equation S1

$$A_{SPME\ Arrow} = 11868454 - 1462428 \times T_{exp} - 889198 \times T_{sampl} - 321278 \times time - 137003 \times T_{exp} \times T_{sampl} + 192962 \times T_{exp} \times time + 40795 \times T_{exp} \times time + 78420 \times T_{exp} \times T_{sampl} \times time$$

Equation S2

Models' validation results were obtained from three independent extractions in the center point of the experimental domain (15°C, 70°C, 22.5 min), as shown in Table S2.

Table S2 – Mean predicted area in the center point of the experimental domain

	A_{SPME}	$A_{SPME\ Arrow}$
Mean predicted area ± confidence interval limits (N=3, α= 0.05)	$(4.6\pm0.3)\cdot10^6$	$(11.9\pm1.8)\cdot10^6$
Mean experimental area ± confidence interval limits (N=3, α= 0.01)	$(5.0\pm0.3)\cdot10^6$	$(14.3\pm0.5)\cdot10^6$
Relative error %	8	17

Considering the aims of the investigation, the models were judged suitable for interpreting the effects of the three factors and their interactions. In detail, it is clear that the principal factor affecting the peak area for both fibers is the exposure temperature which should be fixed at the lower level (10 °C) to maximize the response. Secondly, the sampling temperature also has an effect and should be maintained at the lower level (60 °C) for both fibers. However, this factor is about 50% less important than the former. Regarding the remaining factor, time of exposure, and the two-term and three-term interactions, it is concluded that their effects are much less important than the two temperatures discussed. Therefore, the fibers' exposure time can be selected at the more convenient value for the experiment (i.e., the shorter time of exposure, 15 min). These observations are summarized in Figure S1 and Figure S2, where the relative relevance of the factors discussed is illustrated graphically for SPME and SPME Arrow, respectively.

Figure S1 – Calibration curves for SPME (a) and SPME Arrow (b).

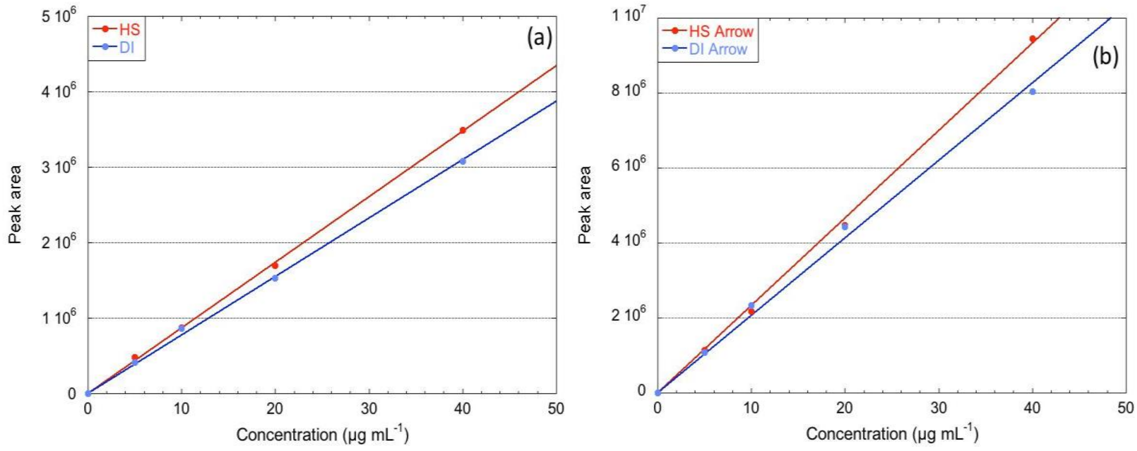

Figure S2 – Normalized quadratic effects plots of the models computed for SPME.

T_{exp} T_{sampl} time T_{exp}·time T_{exp}·T_{sampl} T_{exp}·T_{sampl} ·time T_{sampl}·time

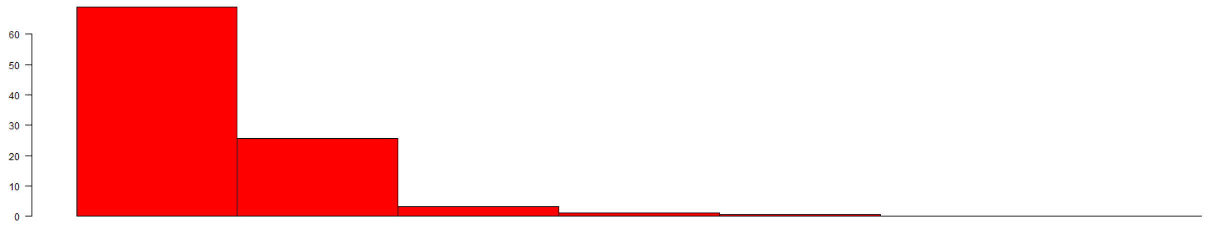

Figure S3 – Normalized quadratic effects plots of the models computed for SPME Arrow.

T_{exp} T_{sampl} time T_{exp}·T_{sampl}·time T_{exp}·T_{sampl} T_{exp}·time T_{sampl}·time

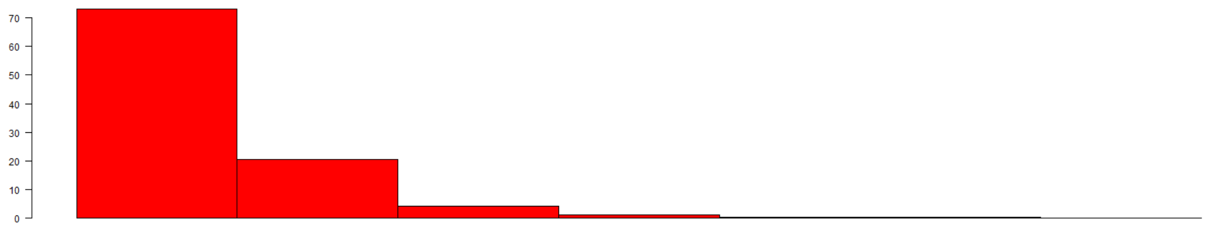

Figure S4 - Calibration curves were constructed using HS and cooling HS for SPME and SPME Arrow.

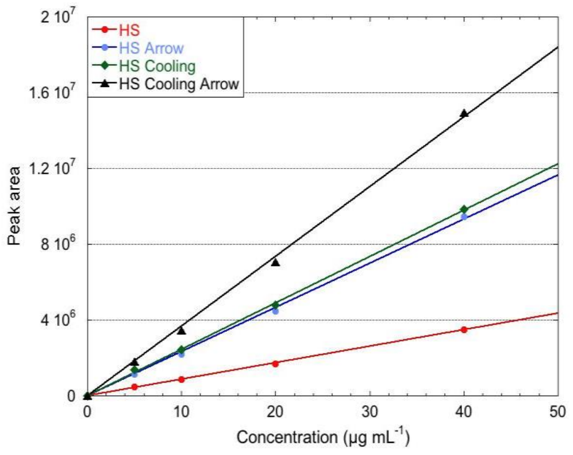

Figure S5 - Calibration curves constructed using cooling MSPME and cooling MSPME Arrow.

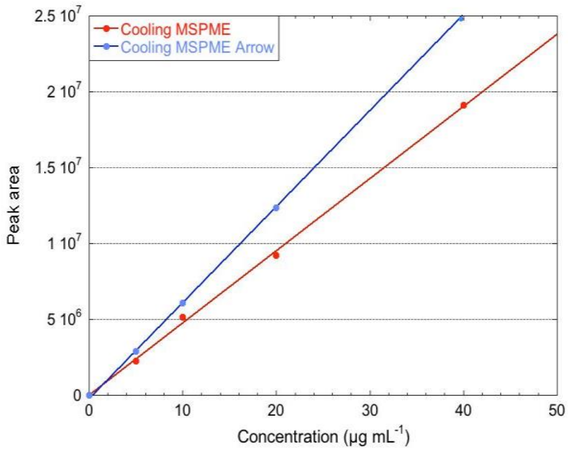

Figure S6 – FA-spiked curves constructed using SPME for green apple (a), plum (b), tomato (c), shampoo (d), and face-wash (e).

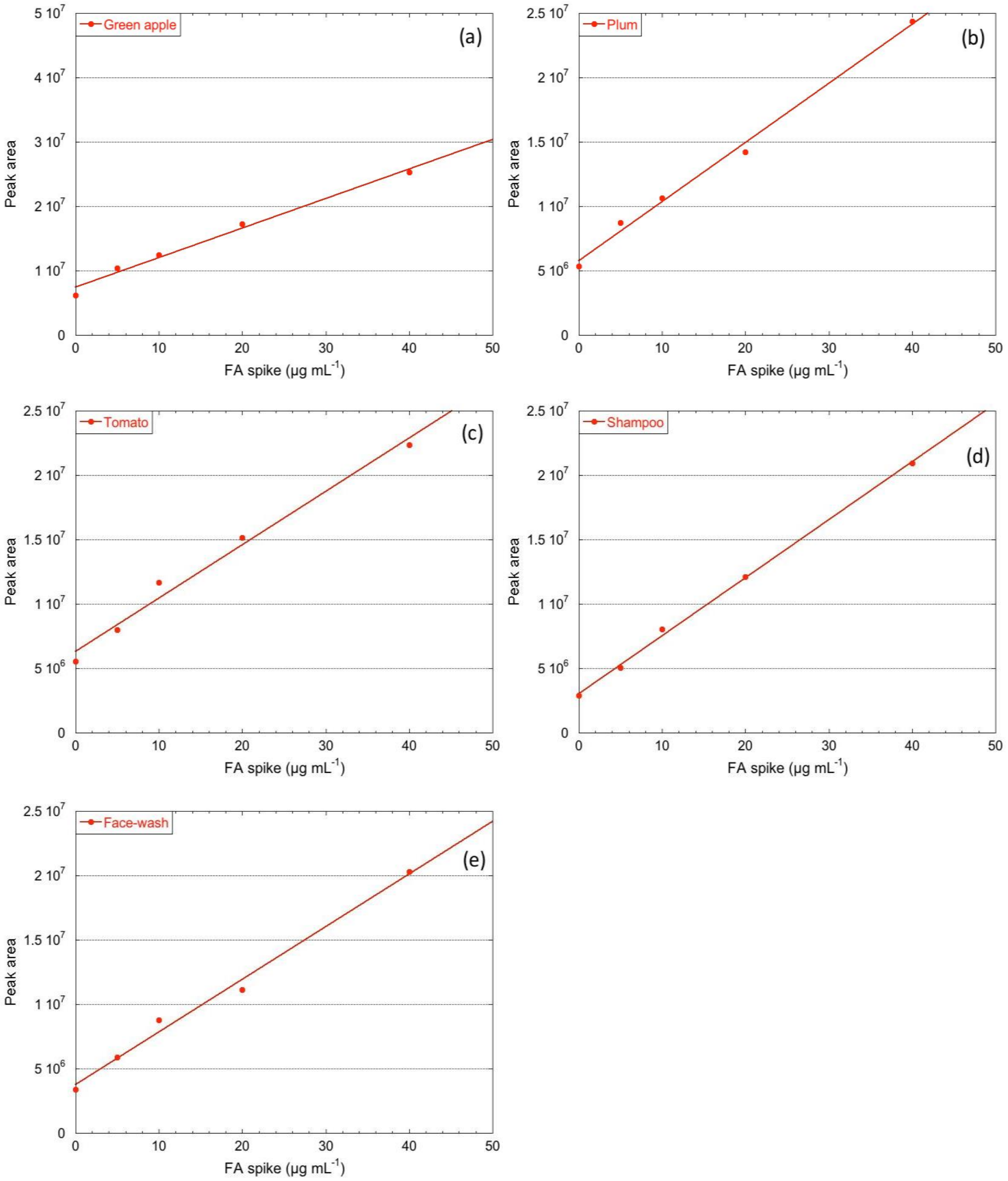

Figure S7 – FA-spiked curves constructed using SPME Arrow for green apple (a), plum (b), tomato (c), shampoo (d), and face-wash (e).

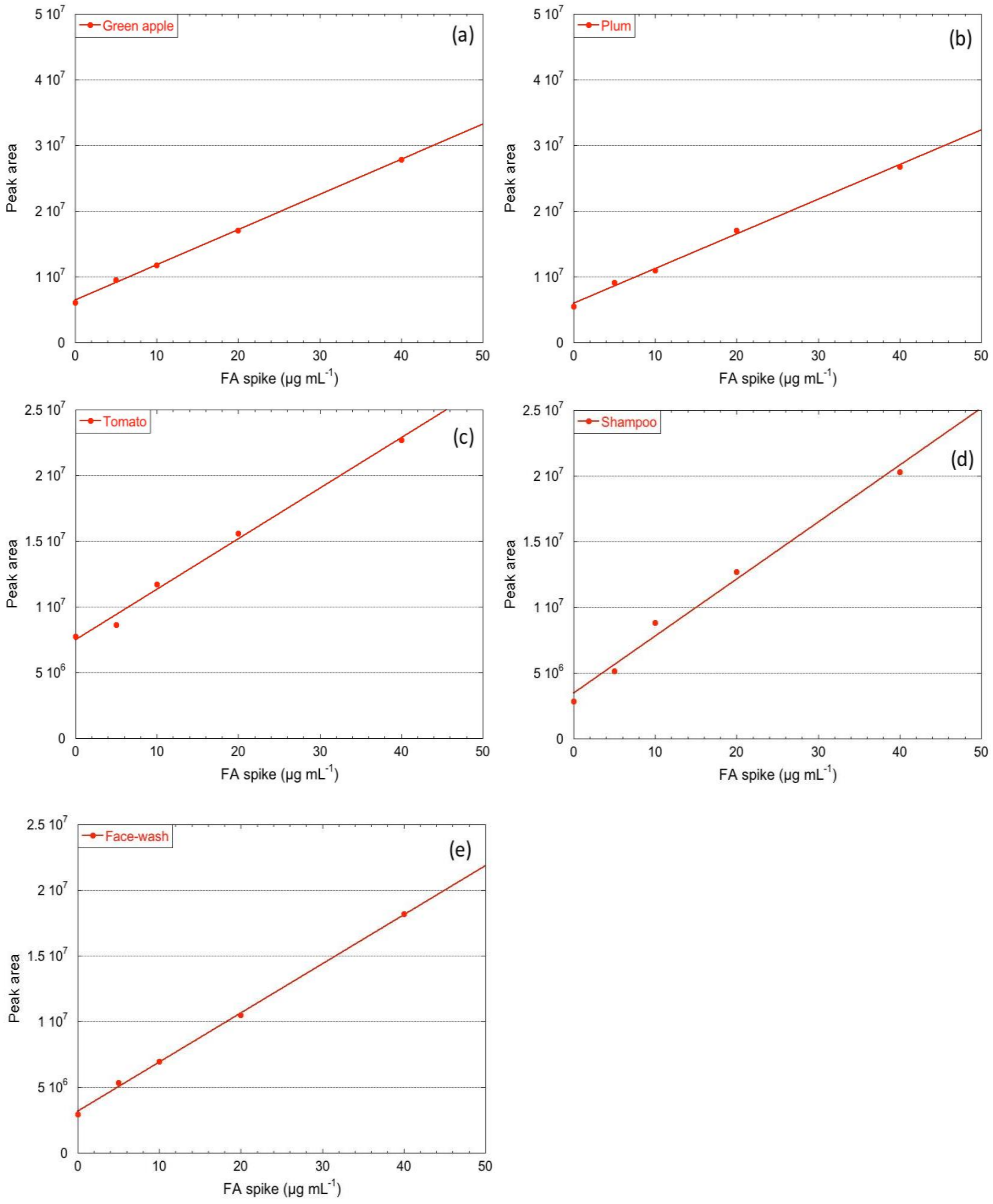

Supplement: Supplementary file 1 [file molecules-28-05441-s001.zip › molecules-2481913-supplementary.pdf]
